# Optimizing simulated interviews and feedback to maximize medical students’ self-efficacy in real time

**DOI:** 10.1186/s12909-022-03512-6

**Published:** 2022-06-07

**Authors:** Shuntaro Aoki, Yayoi Shikama, Kiyotaka Yasui, Yoko Moroi, Nobuo Sakamoto, Hiroki Suenaga, Zunyi Tang, Megumi Yasuda, Yasuko Chiba, Maham Stanyon, Koji Otani

**Affiliations:** Center for Medical Education and Career Development, Fukushima Medical University, Hikarigaoka 1, Fukushima, 960-1247 Japan

**Keywords:** Simulated interview, Simulated patients, Self-efficacy, Feedback

## Abstract

**Background:**

Self-efficacy is crucial in improving medical students’ communication skills. This study aims to clarify where medical students’ self-efficacy is greatest following an interview with a simulated patient and subsequent feedback.

**Methods:**

A total of 162 medical students (109 men, 53 women) in their fourth or fifth year at a university in Japan participated in this study. The degree of self-efficacy in medical interviewing was measured before and after a medical interview with a simulated patient, and after the subsequent feedback session.

**Results:**

ANOVA analysis revealed that self-efficacy for medical interviews was higher after both the interview and the feedback session than before the interview. Among all three time points, self-efficacy was highest after the feedback session.

**Conclusions:**

Feedback following a simulated interview with a simulated patient is important to improve the self-efficacy of medical students when learning medical interviewing skills.

## Background

Because effective doctor-patient communication has been shown to improve both patient adherence [1] and clinical outcomes [2], optimizing clinical communication teaching is a global priority for medical educators in improving the skillset of future doctors.

Self-efficacy, defined as the recognition of one’s ability to successfully perform necessary actions in a given situation [3], has been proposed as a mediator between having confidence and ability to deploy communication skills effectively in a clinical setting [4]. In addition, medical students with high self-efficacy have been shown to have better academic performance [5] and higher metacognitive skills for critical thinking and learning [6].

According to Bandura, as self-efficacy for a particular behavior increases, the likelihood of that behavior occurring also increases, which is further reinforced by success [3]. Thus, it follows that the higher a medical student’s self-efficacy when communicating with patients, the more likely that student is to engage in successful communication behaviors, which, in turn, can positively influence patient outcomes. This is supported by findings of increased patient satisfaction during consultations with medical interns with higher self-efficacy [7]. In contrast, if self-efficacy is low, performance during the interview may be affected, negatively affecting clinical content delivery by increasing patient anxiety and reducing patient confidence in the consultation. Therefore, increasing medical students’ self-efficacy regarding communication skills is vitally important for clinical outcome improvement and professional development.

Simulation training using medical interviews with simulated patients (SPs) represents the mainstream pedagogical approach for teaching patient-centered communication skills to medical students and is supported by peer-to-peer, SP, and teacher feedback [8]. Triangulated feedback provides objective insights and promotes self-reflection into what went well and where improvement can be made, which stimulates behavior change that self-efficacy is well positioned to facilitate. However, while simulation training using SPs has been shown to improve communication skills [9], the mechanisms through which it operates are not well understood, and the relationship between self-efficacy, feedback, and improvement in communication skills remains undefined [10].

To unpack this complex interaction further, it is crucial to understand the impact from the sequence and timing of the interview and feedback on student performance and self-efficacy. Studies have shown how SP feedback following a simulated medical interview results in increased listening scores [11], neurological examination scores [12], and higher self-evaluation scores [13] compared with those who did not receive feedback. Such improvements are also not limited to feedback from SPs, as shown by Brouwers et al., who have demonstrated how feedback from doctors and peers is perceived as valuable by students [14]. However, as self-efficacy was not used as a measurement index in any of these studies, insight into the mechanisms underlying such improvements are limited. To date, the only study assessing the self-efficacy of medical students is that of Pasold et al. [15], who examined self-efficacy in the context of an eating disorder scenario. The students underwent assessments both before and after a didactic lecture on eating disorders, and before and after a simulated medical interview with an SP followed by feedback. While no change was observed in student self-efficacy before and after the lecture, improvements were observed after they underwent a round of simulation training. Because no assessment was conducted between the simulated interviews and feedback, it is unclear which factor contributed toward the improvement. Therefore, it is necessary to evaluate whether the improvement in self-efficacy observed is an effect of the simulated interview itself or of the feedback that followed.

Therefore, in this study, we aim to clarify the point where self-efficacy increases during simulated medical interviews with feedback from SPs, delineating the necessity and timing of feedback, to optimize medical student performance. Such information may inform the format of simulation training with SPs to increase the likelihood of future reproduction of successful communication behaviors in the clinic.

## Methods

### Ethical considerations

This study was approved by the Fukushima Medical University Research Ethics Committee (approval number 30299). Before participation, the purpose and methods of this study, the voluntary nature of participation, the freedom to withdraw from the study, and participant confidentiality protections were explained verbally and in writing. Participants indicated their full and free consent to participate by signing the consent form.

### Participants

A total of 162 students (109 men, 53 women) in their fourth or fifth year of medical education in an urban area in Japan participated in this study. Outcome data was collected between October 2018 and October 2019 during simulation medical interview training. This training formed part of the clinical curriculum that students undertake after passing the pre-clinical Objective Structured Clinical Examination (OSCE), a national Japanese undergraduate medical exam that allows progression to clinical training.

### Program

The participants were divided up into groups of six to eight. The program comprised clinical reasoning and simulation education, with students individually participating in either a clinical reasoning scenario or one of two cases with SPs followed by feedback. The clinical cases comprised a chronic disease risk factor and lifestyle counseling scenario and a breaking bad news scenario requiring giving a cancer diagnosis, with the two cases conducted alternately. Because students participating in the clinical reasoning scenario did not receive feedback, represented a small sample of the total participant population overall and may have increased self-efficacy through participating in the clinical reasoning element itself, they were excluded from the analysis. During the program, students were taught by two doctors with one facilitator supporting the session (a nurse or a clinical psychologist).

**Fig. 1:**
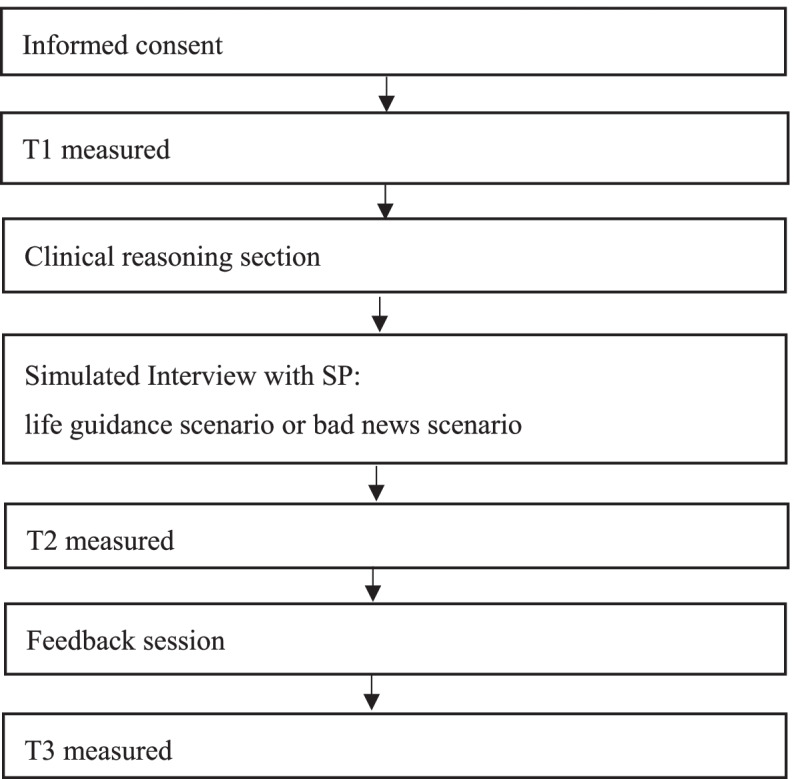
Flowchart of simulated interview with SP

The simulated part of the experience was conducted in two phases: a simulated medical interview with an SP, followed by feedback (Fig. 1). The total session time was about 3 h, comprising clinical reasoning for 40 min, simulated interviews with the SP for 60 min and feedback for 110 min. The simulated interview itself lasted for 10 min with the SP, and was conducted in a private room, with the other students and teachers observing via a monitor in a different room.

During the feedback session, the students first reflected verbally on the experience to the group and received peer-to-peer feedback (including positive and negative aspects of the interview and perspectives on what they would do in the same situation). Next came feedback from the SP (focused on positive aspects of the interview, and feedback on verbal and non-verbal cues) followed by feedback from the teacher (relating to medical points and technical aspects of the communication). Finally, the students reflected a second time on the feedback received.

### Outcome measure

The numerical rating scale (NRS) was used to assess self-efficacy in 11 increments, from 0 (no self-efficacy) to 100 (high self-efficacy) [16]. The NRS scores were the outcome data for this study and chosen owing to prior use for this purpose in Japan, where they have been shown to correlate with OSCE scores [16]. Students were asked, “On a scale of 0 to 100, please rate your ability to do a medical interview,” to assess their self-efficacy while conducting a medical interview. The students circled the number that represented their answers on the NRS. The validity of the degree of self-efficacy on the NRS was confirmed by a visual analogue scale [17]. Self-efficacy was self-measured at three time points: before (T1) and after the simulated interview (T2), and after the feedback session (T3).

### Statistical analysis

Descriptive statistics were calculated for each time point. Repeated measures analysis of variance was performed, with the degree of self-efficacy at each point in time as the dependent variable and time as the independent variable. When the results were significant, a sub-analysis was performed using the Bonferroni method. The effect sizes between each measurement time point were calculated using Hedges’ *g*, and were judged as small at 0.10, medium at 0.50, and large at 0.80 [18]. The ability to conduct medical interviews may be influenced by gender [8] and may be affected by performance differences in the demands of the clinical scenarios themselves. Therefore, we performed two, two-way repeated ANOVAs: one for gender and one for scenario differences between the subjects. If the interaction was significant, a sub-analysis was performed using the Bonferroni method.

## Results

Table 1 shows the descriptive statistics for each time point. The repeated ANOVA showed that the main effect was significant (*F* [2, 322]) = 129.34, *p* < 0.01). Sub-analysis revealed that self-efficacy was higher at T2 and T3 than at T1. Self-efficacy was higher at T3 than at T2. The effect size was small (Hedges’ *g* = 0.31) for T1-T2, large (Hedges’ *g* = 1.23) for T1-T3, and large (Hedges’ *g* = 0.98) for T2-T3. These results indicate that self-efficacy was increased by the simulated interview itself and was further increased by the feedback session.

**Table 1:** Descriptive statistics and results of ANOVA

Self-efficacy score	T1		T2		T3		Time	Time*Group
	*M*	*SD*	*M*	*SD*	*M*	*SD*	*F* value	
All students	42.59	15.18	47.47	17.53	61.48	15.33	129.34 *	-
Gender								
Male	42.66	15.25	46.97	18.18	61.01	16.04	116.03 *	0.28
Female	42.45	15.18	48.49	16.22	62.45	13.85		
Scenarios								
Life Guidance	42.13	16.41	48.65	16.25	62.36	14.70	126.88 *	1.25
Delivering bad news	43.15	13.63	46.03	18.98	60.41	16.11		

*M* Mean score, *SD* Standard deviation, *p* * < .01

We also conducted two repeated measures two-factor AVOVA with either gender or scenario as independent variables. The interactions between time and gender (*F* [2, 320] = 0.28, *p* = 0.76) or scenario (*F* [2, 320] = 1.25, *p* = 0.29) were not significant. Only the main effects of time were significant (Table 1). Therefore, gender and the scenario undertaken by the participant did not affect the above results.

## Discussion

This study aimed to clarify the point where the self-efficacy of medical students’ performing medical interviews increases, i.e., whether this occurs during the simulated medical interview or after the feedback session. We found that, compared with the start of the role play, self-efficacy increased at the end of the role play, before the feedback session had begun. Furthermore, we found an additional increase in self-efficacy after the feedback session, building on the degree of self-efficacy reported at the end of the role play.

Mechanisms explaining this suggest that one’s self-belief in conducting a medical interview increases after gaining actual experience of the interview; thus, effective social skills training includes role play that employs communication skills used in real life [19]. Therefore, we believe that authentic role play stimulated students’ self-efficacy [3], which was then further enhanced through the positive reinforcement gained through feedback. Positive feedback is a social reinforcer, resulting in positive emotions and an increase in the positive cognition that one can perform the same task equally well in the future [20]. Therefore, medical students may have internalized this as, “*I will be able to conduct medical interviews in the same way as I did just now*”, thus leading to an improvement in self-efficacy. This represents a novel finding in the charting of self-efficacy in real time during training and feedback with SPs as part of medical interview training. However, because the setting for our study was a simulated interview, the findings cannot be generalized to patient consultations and require replication in the context of an interview with a real patient.

There are several limitations to our study. One limitation is regarding the effect of behavior modeling which could not be controlled. Modeling increases the probability of an action occurring through observing the consequences of that specific action [3]. As self-efficacy can be improved through modeling and the students observed each other’s interviews and feedback, modeling may have contributed to student self-efficacy. Future research should strive to establish an experimental design that controls for the effects of such modeling, although there are problems specific to such experimental methodologies, such as the inability to reproduce genuine effects obtained in a natural setting. Time, cost, and logistical factors would also need to be considered.

A second limitation lies in not analyzing the four sources of self-efficacy (mastery, vicarious experiences, physiological responses, and social persuasion) [3] with the students. Whilst a measurement such as the one used in the present study is appropriate to uncover the timing-dependent relationship between self-efficacy and medical interviews, further studies measuring all four components of self-efficacy are needed for building a deeper framework.

A third limitation was the clinical reasoning scenario undertaken between the T1 metric and the SP encounter, because participation in the clinical reasoning may also improve self-efficacy.

## Conclusion

Self-efficacy for medical interviewing improves just by conducting a simulated interview with an SP. However, that self-efficacy can be enhanced further through a feedback session after the interview, making feedback an important component to simulation education for increasing self-efficacy, regardless of student gender or interview scenario. Self-efficacy during the medical interview contributes toward ensuring that medical students conduct high-quality, effective medical interviews, enabling subsequent benefit transmission to the patient. Optimizing the sequence and timing of interaction with SPs and subsequent feedback is essential to maximize self-efficacy and increase the likelihood of future successful behavior repetition.

## Data Availability

The anonymized dataset used and/or analyzed during the current study is available from the corresponding author (Shuntaro Aoki, e-mail: s-aoki@fmu.ac.jp) on reasonable request.

## Abbreviations

NRS: Numerical rating scale
SPs: Simulated patients
OSCE: Objective Structured Clinical Examination
